# Dynamic nanoindentation by instrumented nanoindentation and force microscopy: a comparative review

**DOI:** 10.3762/bjnano.4.93

**Published:** 2013-11-29

**Authors:** Sidney R Cohen, Estelle Kalfon-Cohen

**Affiliations:** 1 Department of Chemical Research Support, Weizmann Institute of Science, POB 26, Rehovot, ISRAEL 76100; 2 School of Engineering and Applied Science, Harvard University, Cambridge, MA 02138, USA

**Keywords:** atomic force microscopy, loss modulus, nanoindentation, storage modulus, viscoelasticity

## Abstract

Viscoelasticity is a complex yet important phenomenon that drives material response at different scales of time and space. Burgeoning interest in nanoscale dynamic material mechanics has driven, and been driven by two key techniques: instrumented nanoindentation and atomic force microscopy. This review provides an overview of fundamental principles in nanoindentation, and compares and contrasts these two techniques as they are used for characterization of viscoelastic processes at the nanoscale.

## Review

### Introduction

Understanding and controlling mechanics at the nanometer level is the key to a wide range of cutting-edge topics in science and technology, which range from engineered devices to biological organisms and include novel materials. A number of impressive technologies have been employed in such studies that include, but are not limited to, optical tweezers [1], surface force apparatus [2–3], nanomanipulators [4], electron and other microscopy techniques. Two techniques which have made great advances in the studies of nanomechanics are instrumented nanoindentation and scanning probe microscopy. The versatility and utility of these techniques lies in their capability to measure mechanical response at precise sample locations, in very small volumes and at shallow depths, while monitoring time, depth and force response. The high spatial resolution allows for the determination of local mechanical properties on nanosized objects, and for seeing heterogeneities at the nanoscale, as well as local effects which occur due to a vertical polymer confinement [5]. The time dimension is of particular use in determining the viscoelastic response, which cannot be neglected in analysis of many polymers, biomaterials, and other soft matter.

In the following, we compare and contrast these two point-probe nanomechanical testing techniques. Following a brief review of nanoindentation, we concentrate on the influence and measurement of viscoelastic phenomena. Both experimental and theoretical considerations are included. Finally, a few demonstrative experiments are reported in order to illustrate and critically evaluate the topics reviewed.

### A brief history of point-probe nanomechanical testing

The behavior of materials under controlled stress has enjoyed wide attention over the years. The elastic model developed by Boussinesq [6] and by Hertz [7] is still used today even at the nanoscale under certain limiting constraints. This theory was extended to a range of indenter geometries by Sneddon [8]. However, the pure Hertzian model does not consider surface energies and related adhesion forces, which become significant and may even dominate the overall force behavior at the nanoscale. Several groups considered the effects of adhesion under various contact mechanics models in the 1970s [9–13]. These models analyze the changing contact shapes and stresses that occur when the surface energy and the adhesive forces in the vicinity of the contact are significant. The Johnson–Kendall–Roberts (JKR) theory is appropriate for characterizing contacts of compliant samples with high surface energies, i.e., when there is strong adhesive contact between the tip and the sample [12]. This model balances the elastic energy with the surface energy, expressed as adhesion within the contact zone. It was followed by the Derjaguin–Muller–Toporov (DMT) model, which is applicable for stiffer samples and a lower but non-negligible surface energy, probed by a comparably sharp tip [11]. The DMT model accounts for forces outside the contact zone. These two extreme cases are delineated using the Tabor parameter [10], while the intermediate regime is covered by the work of Maugis [14]. An analytical model has been presented that encompasses all three of these models including the transition region [15].

These, and other important early works [16–19] paved the way for two new point-probe nanomechanical testing devices which were developed in the 1980s – instrumented nanoindentation (INI, also known as depth-sensing instrumentation) [19–20] and atomic force microscopy (AFM, also known by the more general term of scanning probe microscopy, SPM) [21]. These developments facilitated the measurement of mechanical properties of very small volumes of materials, opening new avenues of research. Reducing dimensions to the nanoscale gave birth to new paradigms in mechanical measurements and interpretation: In addition to the increased importance of surface effects such as friction and surface energy, dropping to the sub-optical regime made optical determination of the contact geometry impossible. This led to the need to determine the contact region size from force–displacement curves.

### Fundamental equations and their limitations

The estimation of the elastic modulus from force–deformation curves alone was determined for the Hertzian case by Doerner and Nix who presumed a flat punch geometry to estimate the contact area [22]. The theory was subsequently refined to account for the changing contact area at different points in the unloading curve by Oliver and Pharr (O&P) [23]. The latter developed a nanoindentation model and measurement protocol to quantitatively deduce the elastic modulus and the hardness of materials by loading an axisymmetric indenter into a sample while recording the applied force and displacement. The indenter “area function” is determined by performing this experiment on a well-known material, typically fused quartz. The principle quantities derived from a nanoindentation experiment are elastic modulus and hardness. The former is a fundamental property of the material, which, in principle, can be calculated from bond stiffness, and the latter in turn can be related to shearing bond strengths. The modulus, which was formally defined as the ratio between stress/strain (σ/ε), can be directly computed from a nanoindentation load/deformation curve with the aid of an analytical model such as the widely used O&P approach [23]. This approach uses relations that were derived by Sneddon [8] in order to extract the indentation modulus from the slope of the unloading curve according to Equation 1 [23–24]

[1]
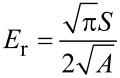


where *S* is stiffness, given by the slope d*F*/d*h* of the unloading curve, and *A* the contact area between the indenter and the sample. *E*_r_ is the reduced modulus which accounts for both sample and indenter Poisson ratio and modulus – υ_s_, *E*_s_ and υ_i_, *E*_i_ respectively:

[2]
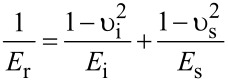


Several depth sensing indentation methods were developed to evaluate *A* [23–25], which allow for evaluation of hardness and modulus without imaging the indentation impression. This model is applicable for flat, homogeneous and isotropic samples, without nonidealities such as adhesion [11–12], pile-up [26–28], and time-dependent effects [29–35].

In practice, this fundamental equation has been extended to samples which do not, in principle, meet the basic requirements. For instance, in heterogeneous materials, sub-micron sized domains can be treated by this analysis when using an indenter with nanometric radius and small indentation depths. Thin, soft films on hard substrates also are well-described by this equation when the indentation depth is limited [36–39].

**Figure 1:**
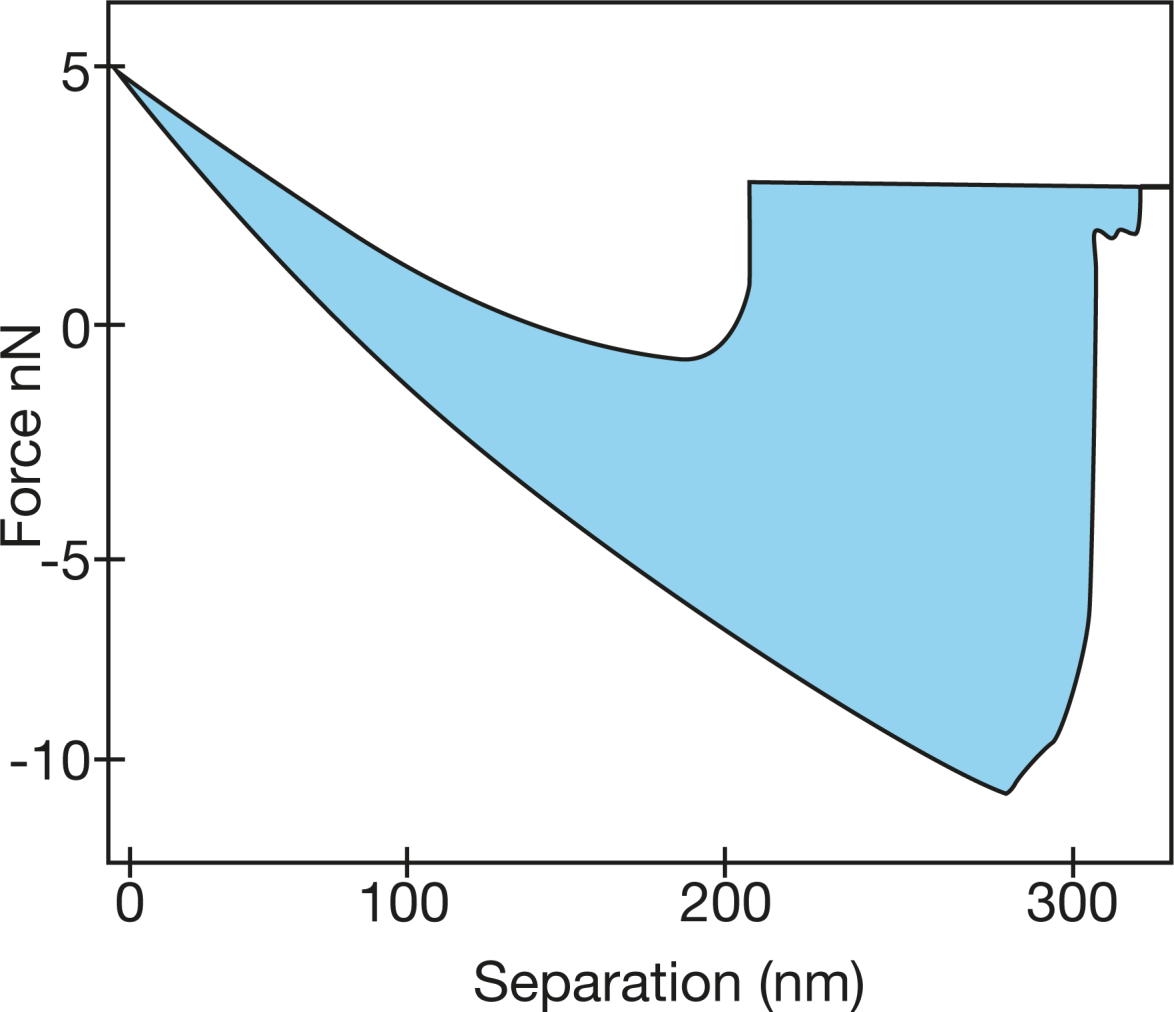
Adhesion-induced hysteretic negative unloading force observed on indenting PDMS (4% cross-linked) by AFM with a pyramidal silicon nitride tip of force constant 0.05 N/m. The colored area represents the hysteresis between ingoing- and outgoing traces and defines the energy loss.

Whereas limiting indentations to the nanoscale justifies ignoring some types of inhomogeneities, the influence of small, intrinsic adhesion forces is enhanced. The presence of capillary and adhesive forces changes the contact profile and modifies the force acting between indenter and sample during pull-out. This behavior is highlighted by a significant attraction between tip and surface upon pull-out seen as a hysteretic negative unloading force (Figure 1). Accordingly, the contact radius depends on the thermodynamic work of adhesion, ∆γ, considered in the JKR theory.

Ebenstein and Wahl examined several ways of calculating the modulus for real experimental data, in order to handle the inaccessibility of some necessary parameters. For instance, since the true contact area is rarely known, for the JKR relation [40]:

[3]
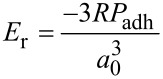


The contact radius at zero net force, *a*_0_ as well as the adhesive force *P*_adh_ can be found by curve fitting. Typically unloading curves are preferably taken for analysis to avoid the plastic deformation that is present during loading. In AFM this procedure may lead to erroneous results: Since the AFM experiment does not control the load, but rather the displacement, the tip–surface system has an additional degree of freedom because of a compliance of the cantilever, which leads to a more stable contact during unloading. Hence, the fit to the JKR model, which presumes load control, is invalidated [41].

For the DMT model the interaction is described by:

[4]
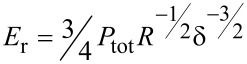


where *P*_tot_ is the total force, including *P*_adh_, and δ the sample deformation.

The Tabor parameter, which is used to distinguish between JKR and DMT conditions is given by [10,40]:

[5]
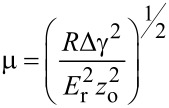


When µ is less than 0.1, the DMT theory is applied and when μ is greater than 5, the JKR theory is used [40]. The intermediate regime between these two extremes is covered by the work of Maugis [14]. The choice of the model (JKR, DMT or Hertz), that is used for the reduced modulus calculation then requires some a-priori knowledge of the material or preliminary investigations as will be further developed below.

### Instrumentation

**Figure 2:**
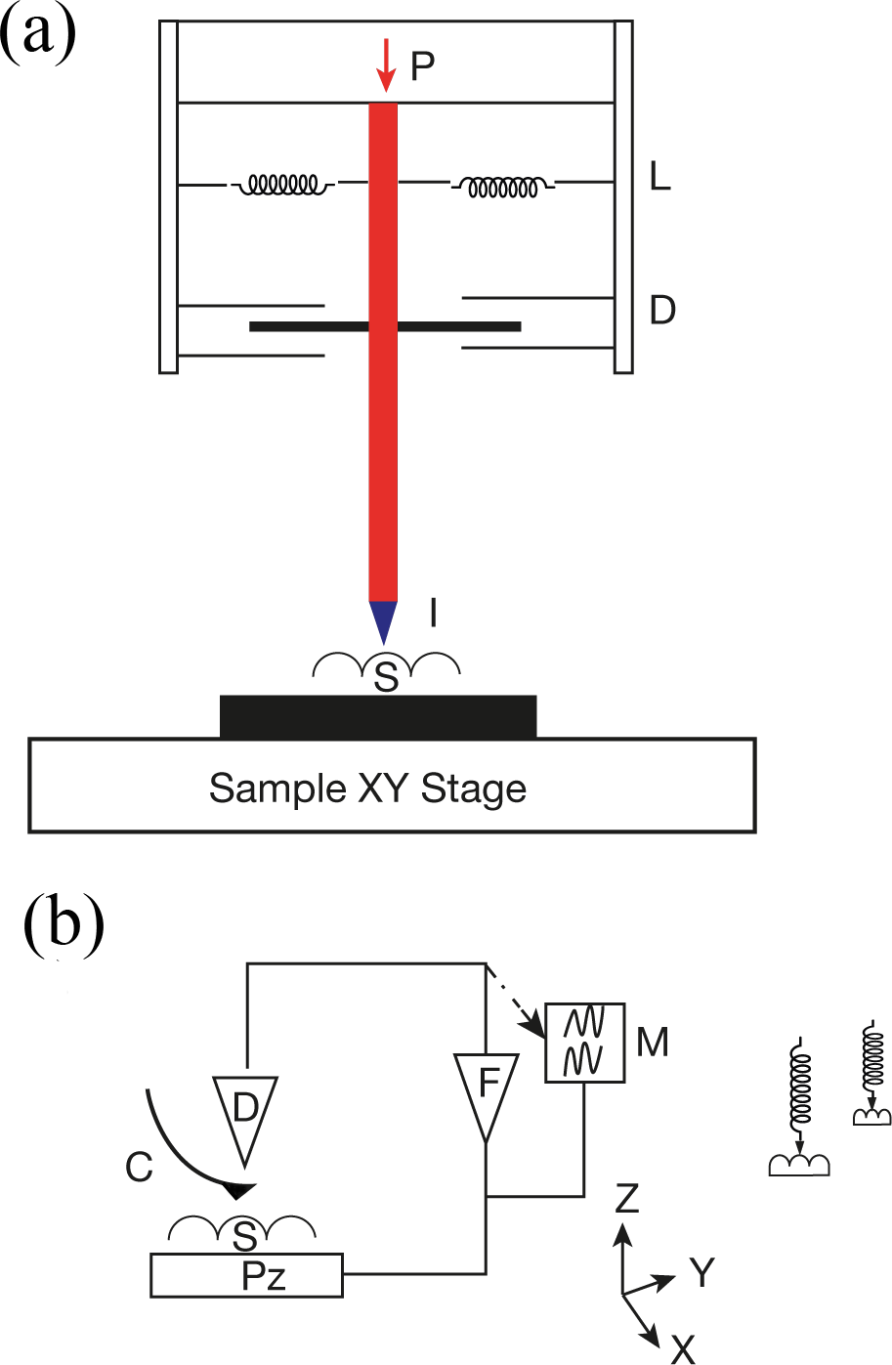
(a) Schematic of instrumented nanoindenter, including P - calibrated force; L - support springs; D - Position sensor; I - indenter tip supported by shaft; S - sample. (b) Schematic of AFM showing Pz - piezoelectric motion transducer; S -sample; C - integrated cantilever and tip; D - cantilever motion detector; F - feedback; M - monitor for display. To right the working of AFM is shown schematically: extension of spring changes before and after applying load. From [42].

Schematics of INI and AFM instruments are shown in Figure 2. Table 1 gives a comparison of their capabilities and characteristics. For INI, a calibrated force is applied to the indenter tip, which in turn is constrained with a vertical spring. The lateral spring constant can be considered infinite in the standard configuration, and the indenter motion is confined to the plane perpendicular to the sample. The vertical indenter displacement is measured independently, providing nm-level sensitivity. The accurate determination of the contact point then allows for a direct determination of the indentation depth. Indentation placement is directed by an optical view, or in some cases the indenter tip itself is used to make a higher resolution profiling scan of the surface to enable a placement in the tens of nm range.

**Table 1:** Comparison between instrumented nanoindentation (INI) and atomic force microscopy (AFM).^a^

characteristic	INI	AFM

vertical spring constant	100 N/m	from <0.01 N/m to over 500 N/m
lateral spring constant	10^5^ N/m	10–1000 N/m^b^
lowest fundamental resonance	20–500 kHz	several thousand kHz
displacement sensitivity	1 nm	0.05 nm or better
load sensitivity	10 nN	<0.05 nN^c^
dynamic range of force	10^8^	10^3^
nm-scale imaging	nonexistent to fair	excellent
bandwidth	0.001–100 Hz	1 Hz – several kHz
temporal stability^d^	good	fair

^a^Parameters given here are typical and may vary from instrument to instrument, ^b^Typically 2 orders of magnitude greater than flexural (normal) spring constant, ^c^Depends on cantilever used in measurement. ^d^Sensitive to instrumental design: environmental control can improve this.

For AFM/SPM the situation is somewhat different. A calibrated displacement is applied to the base of the probe or to the sample, and this motion is transduced into force by a flexing of the cantilever beam that holds the probing tip. The degree of flexure is measured, usually by optical means, and the force is obtained with knowledge of the cantilever stiffness and of the measured degree of bending. The sample–tip motion is actuated by piezoelectric elements, which can be linearized by closed-loop control. The cantilever beam is usually oriented at an angle to the surface, which results in some tangential force being applied in addition to the normal force. A tangential motion along the long cantilever axis can lead to additional flexural bending [43], while a force orthogonal to this direction results in a sideways torsion with the ultimate torque being moderated by the tip length [44]. Thus, the choice between INI and AFM typically involves a trade-off between a more reliable characterization of force and a larger dynamic force range for the former and a better force and displacement sensitivity and a superior imaging/placement for the latter [45–47]. For soft materials, in which an insensitivity to the initial contact can lead to a severe underestimation of the contact depth in INI, the enhanced sensitivity of the AFM carries some distinct advantages. However, even though AFM has been successfully used to probe mechanical properties on the nanoscale, there are a number of drawbacks, which make the quantification of the mechanical properties challenging. Many of the factors discussed in previous reviews over the past decade are still issues today [48–51]. Most notable of these are the implicit assumptions of linear elasticity, which require the contact radius and the indentation depth to be much smaller than the indenter radius, and the absence of tangential stress so that forces are restricted to the surface normal – all these are difficult to maintain in AFM.

### Dynamic nanoindentation

#### Background and relevant models

**Figure 3:**
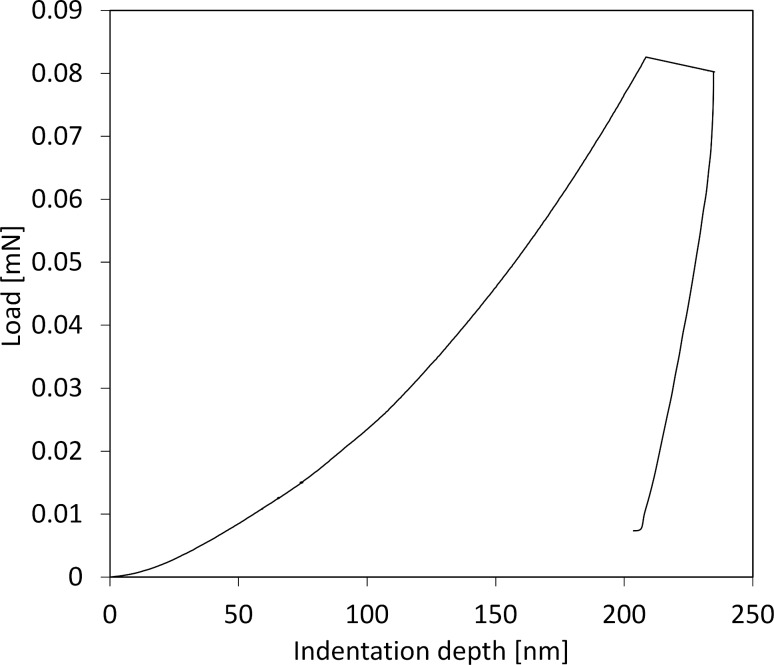
Load-vs-displacement curve taken by nanoindentation with a Berkovich indenter tip on a polyurethane sample showing negative stiffness, seen as the outward bulging of the curve, which develops in the unloading (right branch) of the curve, because of creep.

Time-dependent phenomena, i.e., when the material strain is not synchronous with the force or displacement applied to the perturbation stress, have a strong influence on the load-vs-deformation curves. Since there is a time lag between a change in applied stress and the response of the material, the deformation response still “remembers” the increase in stress during a hold at peak load or even during unloading. This leads to a “nose” in the curve (as shown in Figure 3) and, in extreme cases, to an apparent negative stiffness. This phenomenon will depend on the rate of change of the force. Computational approaches exist that modify the Sneddon contact mechanics model to remove the time-dependent effects of viscoelastic materials [52–53]. Time-dependent compliance is ubiquitous and can appear even in quite hard ceramics as result of the rearrangement of point defects [54]. Under some conditions, the nose can be due to viscoplasticity [55]. By choosing working conditions that avoid viscoplastic deformation during unloading, it may be possible to apply the O&P model with consistent results [56]. However, there is still a need to utilize analytical approaches that are directly suited for viscoelastic materials. Many examples of such work can be found in the literature. Since the classical solutions assume equilibrium conditions, which do not strictly hold when there is a viscoelastic response, numerical approaches have been developed [57–58]. The variety of analytical approaches can be justified by different considerations that must be made for different classes of materials as has been recently reviewed [59].

The wide variation in experimental protocols that is applied for dynamic studies is a natural outcome of their diversity. In order to put some order to these methods, it is necessary to understand the basics behind the physical phenomena. Here, we will limit the discussion to linear viscoelastic behavior, which means that the strain depends only on time and not on the magnitude of stress. This holds when the stress is kept small. The dynamics can be experimentally studied by several means, the most common being summarized here [60]:

1. creep relaxation, in which the indenter is rapidly brought to a given force/stress and the change in strain required to maintain this situation is monitored.
2. stress relaxation where the indenter is brought to a given deformation/strain and the stress required to maintain it is monitored.
3. periodic variation of stress, usually sinusoidal, at a given frequency *f*, equivalent to a transient experiment at time *t* = 1/2π*f* = 1/ω.

**Figure 4:**
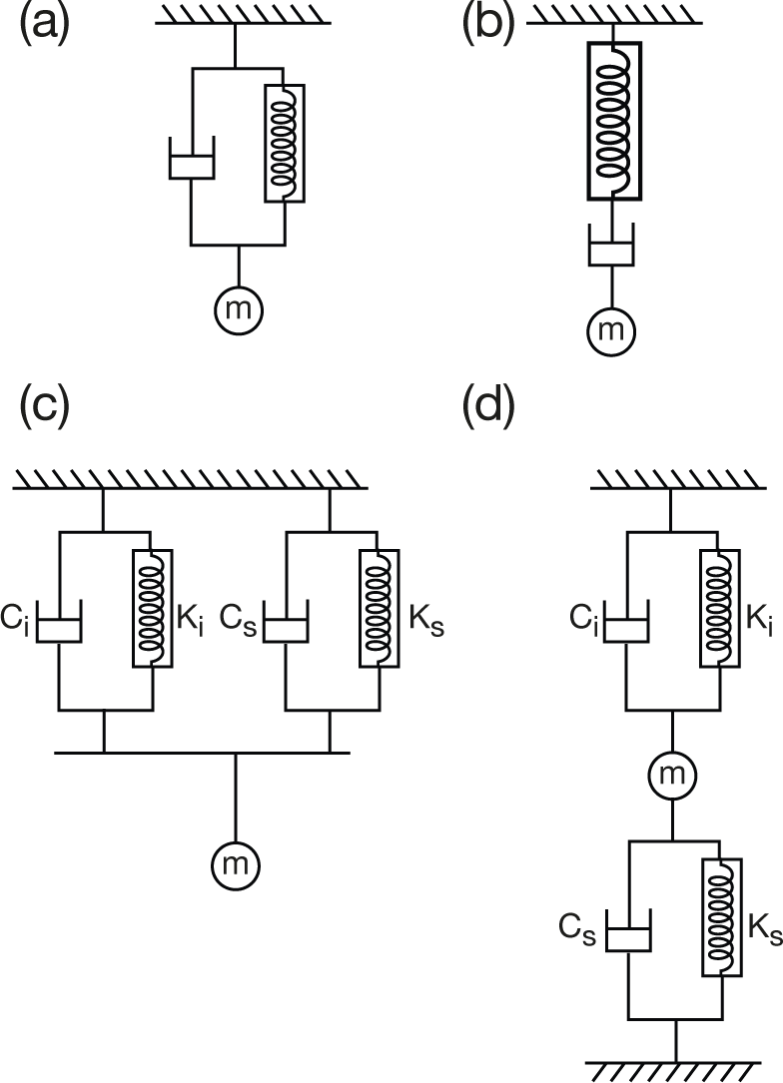
Fundamental models for a viscoelastic mechanical system. (a) Voigt model (b) Maxwell model (c) Generalized Voigt model with 2 components (d) Voigt–Kelvin model.

To formulate the time response in terms of well-understood mechanical elements, the viscous component is modeled most simply as a combined spring and dashpot either in parallel (Voigt model) or in series (Maxwell model), shown in Figure 4a and Figure 4b. A comparison of these two models, as well as the resulting constitutive equations can be found in the book of Shaw and MacKnight [61]. These models address the fact that the mechanical behavior of a viscoelastic material cannot be described by either a simple spring or by a viscous element. In general, the Maxwell model is more appropriate to a viscoelastic fluid and the Voigt model a viscoelastic solid. In comparison, these models yield the same results, except that the Voigt model cannot describe a stress relaxation experiment because the dashpot would develop a singularity in force with step change of strain.

To illustrate these differences, for load control as it is used in INI creep relaxation, the Voigt model, Figure 4a, can be applied, but for nanoindentation stress relaxation as it is conveniently done in AFM, the Maxwell model (Figure 4b) is more appropriate. Whereas these simplistic models give a general means to quantify the physical phenomena, they do not encompass the complexity inherent in a real system. Therefore, combinations of Voigt and Maxwell components are often used to better approximate reality. These could be a series of constitutive elements connected in parallel, or in series, depicted in Figure 4c and 4d, respectively. A combination of Voigt elements connected in series is known as the Voigt–Kelvin model. This model and its modifications are widely used for nanoindentation creep relaxation analysis due to the suitability of the Voigt model for solids as mentioned above [62–64]. Combinations of the different models are also used. A comparison of three different models (Maxwell, Voigt–Kelvin, and combined Maxwell and Voigt–Kelvin) revealed that the latter gave the best fit to experimental data and also displayed a predictive power for experimentally-obtained indentation curves [65].

Classically, in the DMS (dynamic mechanical spectroscopy) technique, a macroscopic sample is subjected to a modulated stress and the strain is recorded [66]. The energy released by the relaxation of a polymeric chain generates a phase shift between the harmonic stress and strain, which is in turn used to express the dynamic elastic moduli as defined above. This shift is frequency-dependent, as the various modes of internal friction of the polymer are excited at distinct characteristic times. In order to probe the viscoelasticity at the nanoscale analogous techniques are applied. An interpretation should consider that the measured phenomena could be different at the nanoscale for the general reasons already discussed and more specific properties of polymers such as issues of confinement [5,67].

We first consider the modulation experiment for which either the stress or the strain could be modulated. In this case, stress and strain will exhibit a phase difference designated as angle δ and the modulus can be now expressed as complex modulus E*:

[6]
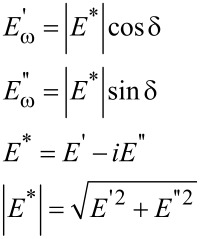


Here, *E*′ is the storage modulus, which measures the energy stored during one oscillation cycle, and *E*″ is the loss modulus, which measures the energy dissipated during an oscillation cycle.

The phase lag, referred to as loss tangent (tan δ), arises from any of a number of molecular-level lossy processes such as entanglement, slip or friction between the monomer units. Although the phase lag is not amenable to a direct theoretical interpretation, it is relatively easy to be determined accurately and provides useful qualitative information. Furthermore, tan δ takes on characteristic values, e.g., approximately 1 for amorphous polymers in the transition zone, and 0.1 for glassy and crystalline polymers [60]. Most importantly, it does not require any knowledge of the contact area and it can be used to clearly identify phase transitions.

At the micro-level, the thermodynamic state of the polymer can be related to a molecular motion at different hierarchical levels – from the cooperative motion of entire chains through short hops of individual segments and finally to internal rotations and vibrations of the component molecules. Polymers exhibit several phase transitions that can be correlated with the relaxation at characteristic frequencies [66]. The loss modulus will vary over a wide range of frequencies and show peaks at specific temperatures and frequencies that correspond to phase changes. These changes can be effected both mechanically by a change of frequency, and also through internal thermal motion. A related approach to understand the molecular dynamics is thus by varying the temperature [68–69]. The correspondence between temperature and frequency is embodied in the temperature–time superposition [70]. In general, increasing the temperature induces a molecular relaxation that leads to an increased phase lag between stress and strain. The loss modulus, which reflects the viscous damping of the sample, then increases with temperature. Concomitantly, the storage modulus is reduced since molecular relaxation loosens the molecular bonds.

#### Experimental aspects of the dynamic response measurement

Some caveats should be applied in comparing experiments made under different conditions: The creep response depends on the tip shape, and results obtained by using the common Berkovich indenter show an apparently more compliant sample than a spherical tip [35,71]. Investigation of dynamic elastic contacts showed that the frequency of oscillation strongly influences the contact radius [33]. Furthermore, the creep compliance and the time-dependent shear modulus can vary with the ultimate force applied because of a deviation from linear viscoelastic behavior [30].

Because of their small size and the fine control over force and displacement, the point probes are particularly amenable to dynamic loading, in which a small modulation of several nanometers is superimposed on the quasistatic loading curve, and the displacement amplitude and phase angle between the applied modulated force and the corresponding modulated displacement are measured continuously at a given excitation frequency [72]. Detailed explanations of force modulation techniques and analyses can be found in the literature [20,73]. Briefly, the modulated force *P = P*_0_ sin(ω*t)*, results in a displacement oscillation at the same frequency expressed by *h*(*t*) = *h*_0_ sin(ω*t* − φ)*.* The dynamic in-phase and out-of-phase equations can be re-written in terms of experimental observables as follows [74]:

[7]
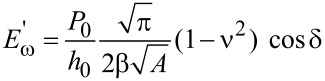


[8]
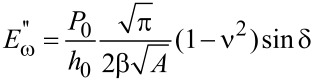


where *P*_0_ is the steady-state modulated load amplitude, *h*_0_ is the resulting modulated displacement amplitude, and δ as defined above is the angular phase shift between the applied force and the measured displacement. The contact area *A* is not an observable, but can be derived by calibrating the indenter. It has been noted that these relations hold strictly only for shear between two parallel plates and the application to nanoindentation experiments should be used with caution [75].

By using the dynamic model shown in Figure 4c, an analytical solution for the resulting displacement amplitude, *h*_0_, and the phase shift, δ, can be derived. In AFM, the modulation may be applied at the tip or at the sample, which will lead to different analytical solutions [63]. Here, the solution is given for force modulation applied to the tip, as is typical for INI [64,73]. Thus, the measured displacement amplitude *h*_0_ induced by the modulated force amplitude *P*_0_ is:

[9]
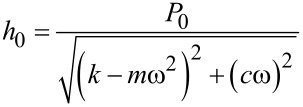


and the measured phase shift between the applied force and measured displacement is related to sample and instrumental parameters by:

[10]
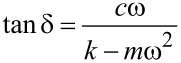


[11]
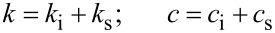


where *c*_i_, *c*_s_ are the damping coefficients of the air gap in the displacement transducer and sample, respectively, *k*_i_ and *k*_s_ instrumental and sample stiffness, and *m* the indenter mass. Typically, no correction is made for the additional moving mass of the contact since it is insignificant relative to the indenter construct. In the case where this assumption would no longer be valid, an effective mass should be used in the equations together with effective *k* and *c* in order to avoid an overestimation of *c* and hence of *E*”.

After calibration to determine *m*, *c*_i_, and *k*_i_, the sample-specific values for *E*′ and *E*″ can be obtained as shown in Equation 12. These equations also illustrate how the damping coefficient can be understood as the out-of-phase counterpart to the in-phase stiffness giving the storage modulus. This leads naturally to expression of tan δ as the ratio of *E*″/*E*′.

[12]
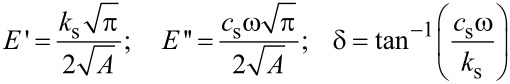


Despite the fact that for the configuration of nanoindentation experiments, the basic assumptions that underlie such models are not strictly satisfied, they yield reasonable results relative to classic rheological studies and other macroscopic measurements [35,75–78].

Creep relaxation, which was introduced in the previous section, is also used to determine time-dependent phenomena. Although most studies invoke the loss modulus *E*″ and a viscosity coefficient η, Yang et al. assign three contributions to the creep [79]: the elastic deformation, analogous to the deformation used in Equation 1, the viscoelastic deformation controlled by an exponential term, and the viscous component η.

In the creep experiment, the probe tip is pushed into the material at a fixed load *P*_I_ and an initial depth *h*_I_*.* The force is held constant by the system feedback throughout the creep time and the creep is detected as change in position required to compensate for the relaxation and to maintain constant force. Relaxation of the viscoelastic material then results in an increased indentation depth. In principle, the creep displacement can be directly read from the experimental displacement curves. For INI this would be the displacement sensor reading of the indenter position. For AFM it would be the *z*-piezo extension, which is ideally monitored by a linearized sensor. However, thermal drift and piezo creep can also contribute to the apparent displacement, thus they must be minimized and/or measured and corrected for. This can present a challenge, particularly in AFM, which largely relies on piezoelectric motion transducers. It should be noted that a *z*-sensor, which is used to linearize the *z*-motion, cannot distinguish between creep and thermal drift. In light of this discussion, additional differences between AFM and INI can be added to those mentioned above: the time resolution of the measurement, which is related to the inertia of the system, and the drift/creep characteristics. These are noted in Table 1 as bandwidth and temporal stability.

For a given creep time *t*, the corresponding displacement into the surface *h*(*t*) is measured experimentally. The creep behavior can then be modeled following [79] and [80]:

[13]
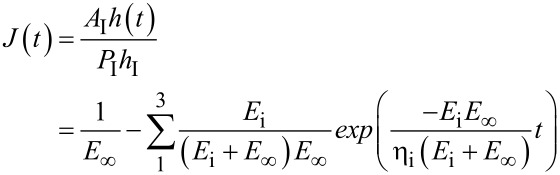


with *h*_I_ being the initial indentation and *h*(*t*) the indentation at time *t*. *A*_I_ (initial contact area) and *P*_I_ are known or measured; Such curves are then related to different relaxation modes of polymers and the frequency spectrum of internal modes by models such as Voigt–Kelvin, for which the model in Figure 4d is extended to *n* intrinsic units. The applicability of different mechanical models to a unique mechanical system is an established mathematical concept that was recognized in the interpretation of polymer systems (see [60], pp 16 and 17). Sun and Walker applied the Zener model, with these elements connected in parallel, to model creep in a number of different polymer systems. They found that 1–3 elements were required depending on the polymer studied. Ideally, each such element, with its characteristic time, represents a given transition in the polymer. As usual, over-interpretation should be avoided, and one should always recall that the simplified or even complex mechanical models are describing a substantially more complex molecular system.

#### Dynamic AFM probe nanoindentation

Boasting the advantage of wider bandwidth, smaller inertia of the system, better lateral resolution and more sensitive force detection, AFM provides some interesting opportunities for monitoring the dynamics in nanoindentation. Operating the AFM under dynamic mode has distinct advantages in reducing the sample damage, particularly for delicate samples [81–82]. Also, the volume needed for probing is reduced even further, which allows for the analysis of small areas and thin films down to a single monolayer [83]. A recent example is the application of AFM imaging together with the mechanical measurement to give a detailed insight on cellular membrane mechanics, which is only meaningful when the viscoelastic response is accounted for [84]. The bandwidth advantage has been extended to the MHz range, allowing an access to higher harmonics [85–89]. This provides several advantages: Higher harmonics can be exploited to separate the mechanical measurement from the topographic feedback, the signal-to-noise ratio can be improved, and the accessible dynamic force range is enhanced since each harmonic is associated with its own characteristic spring constant. Investigation of a material over a wide range of frequencies also gives a sharper topographic contrast since some materials, which may yield under the tip force, are unable to respond at high modulation frequencies and thus appear to be stiffer. The inclusion of multiple, higher frequencies, also allows for a full characterization of the highly nonlinear cantilever dynamics [81,90]. Nonetheless, the application of such techniques still requires the knowledge of contact geometry, an assumption of some contact model and/or force potential, and in some cases the input of some parameters of the material. Thus the inherent fundamental limitations of quantitative nanomechanical testing must be accounted for. New noncontact techniques allow for the monitoring of the entire force profile while starting at noncontact positions. The deconvolution implemented to convert the experimentally observed frequency shift/amplitude change to a force can also introduce some uncertainty [91]. Single-frequency techniques are still more readily accessible in most laboratories. Dynamic imaging modes that are commonly used in AFM provide the phase information, typically as an image channel measured and displayed simultaneously with the topographic image. The phase shift is interpreted as giving an estimate, generally qualitative, of the energy dissipation [92–93]. Nonetheless, there are many contributions to such phase contrast including the changing tip–surface contact area as the tip scans the sample. Each case must be modeled differently.

Theoretical studies exploit phase and amplitude data together to identify and quantitatively measure the different dissipation processes [82,94]. One caveat arising from the modulation techniques is that the phase lag signal carries information on additional dissipative processes other than viscoelastic energy dissipation, such as surface adhesion and capillary forces [95]. “On-the-fly” measurements of dissipation, which integrate the area under the hysteretical force–distance curves as depicted by the blue-shaded region in Figure 1 can also include these effects of adhesion and thus cannot be unequivocally assigned to viscoelastic processes in the material. Such effects are critical issues for polymers. For this reason, equating phase contrast with a viscoelastic effect would be misleading in many cases. Calculations by Garcia and coworkers succeeded in separating and quantitatively reproducing different contributions to dissipation [96]. This work underscores the importance of including the contribution of the oscillating cantilever to the overall mechanical response.

Eastman and Zhu show that the adhesion forces depend strongly on the surface energy of the tip, and on the wettability of the tip surface in a humid environment [97]. In ambient conditions, the surface of a polymer is likely to be covered by a thin layer of water that is sufficient for increasing the capillary and possibly van der Waals interaction. Any additional adhesion will act to increase the hysteresis in the force–distance curve and thus complicate the application of simple mechanical models.

In addition to these environmental effects, the instrumental contribution to damping must be accounted for. These inherent instrumental properties include those of the spring (cantilever), of the electronics, and of the piezoelectric transducer. For INI, careful calibration protocols have been described to account for these [64,73]. For SPM there is no unified approach to this procedure, yet. Such calibration requires knowledge of the frequency-dependent amplitude and phase shift and is critical for accurately evaluating the stiffness and damping coefficient of the tested material. For dynamic INI, the modulation is applied at the tip–sample contact. In dynamic AFM operation the displacement may be applied to the base of the cantilever or to the base of the sample, in which case the cantilever spring acts in series with the tip–surface compliance [20,73]. A very different response is obtained when the modulation is applied at the tip–sample contact [98]. Burnham et al., in an analysis of the mechanics of dynamic AFM contact, described the various modes, in which the AFM can be used to study energy dissipation [63]. They split the possible operation modes into three categories: force modulation, sample modulation and tip modulation. They found that a proper choice of measurement category and associated frequencies is needed for different types of samples.

The system response for a typical AFM measurement can be calculated from Equation 12, following the setup of Figure 4d. In this case the total stiffness *K* and damping *C* differ from Equation 11 as follows:

[14]
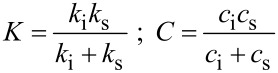


The instrumental contributions to stiffness and damping, *k*_i_ and *c*_i_ respectively, are determined by an independent calibration. Then, from the measured system response, the stiffness and damping of the sample can be extracted. Clearly, this procedure entails a careful calibration of the instrumental damping both for INI and AFM.

Researchers have wrestled with the best way to measure the dynamics for two decades. One of the earliest attempts to measure the dynamic modulus by AFM was performed by applying a modulation directly to the sample *z*-piezo [99]. In this early work, the instrumental phase shift was removed by conducting a comparative measurement on a stiff, clean surface. By modifying the AFM setup, Hutter et al. induced small oscillations to the deflection signal by inserting the modulation directly to the feedback loop to generate a compensatory oscillation of the sample *z*-piezo [100]. This approach is proposed as easy to implement, and allows for the measurement of the dynamic modulus while imaging the sample. This setup provided a quantitative measurement of the viscoelastic properties of PVA fibers. Also here, the instrumental phase response was assessed by a measurement of a perfectly rigid sample. McGuiggan and Yarusso calibrated the instrumental phase shift on a hard surface. They pointed out that this method ignores slip that may occur at the surface [101]. They furthermore note that most models do not include adhesion hysteresis, an unknown tip shape, and other factors. They chose to report their data as tan δ since the ratio between loss and storage modulus cancels out the unknown terms.

Minary-Jolandan and Yu also related to the potential errors when the instrumental phase shift is measured on a hard surface and noted that it may lead to large errors on rather compliant samples, which exhibit only small phase shifts [102]. They proposed a method to remove the offset phase directly on the sample of interest at zero contact force, which provides an accurate in-situ calibration of the instrument. This method accounts for all factors contributing to the phase shift except for those caused by the compression of the sample under the tip. Therefore, this method would not work on a soft polymer brush with ill-defined interface.

For the resonance modes, system response and phase shift are non-negligible in air. Yuya et al. removed the internal beam damping by evaluating its behavior suspended in air [103]. A known reference was used for calibration, allowing them to measure storage and loss modulus with an ultrasonic contact technique utilizing the first three flexural modes. For measurements in any fluid, including air, there will be a drag force on the bulk cantilever. In liquid, this drag force is very significant. Mahaffy et al. recorded this force in an aqueous environment as the tip approached the surface, but before it made contact and thus comprised a noncontact measurement of the phase shift [104]. A combination of the two corrections was made in a study of viscoelastic behavior of cells [105]. In this work, the hydrodynamic drag of the cantilever was measured at varying heights above the surface, and in addition, the phase response of the AFM piezo was measured in contact with a stiff cantilever probe on a hard surface in air. These few examples prove that there is no accepted standardized protocol for the characterization of the intrinsic phase shift, even though the use of a rigid substrate as reference is quite widely used.

The point-probe techniques also lend themselves to measurements that are not necessarily based on AC methods for the characterization of viscoelastic materials. In many dynamic processes it is found that more energy is required to separate two surfaces than is released when they come into contact. This is usually manifested as a hysteresis between the loading and the unloading curves in force measurements. The fine resolution can be achieved with noncontact AFM allows for a quantitative detection of dissipation that involves the formation and the breakage of weak intermolecular bonds in an organic molecule [106]. However, for many practical cases, the separation entails energy dissipation in the bulk material – generally a viscoelastic/plastic deformation, as well as capillary and adhesive forces.

The analysis of AFM force–distance curves of polydimethylsiloxane (PDMS) showed a strong influence of the measurement conditions such as the loading–unloading rate and the dwell time, as well as intrinsic material properties like the crosslinking density and chemical surface modifications [80,107–109]. The viscoelastic response has been studied both through hysteresis in the loading–unloading portion of the curve [110–111], and in the adhesive pull-off segment [80,109]. Analyzing the results in this fashion allows one to distinguish between the bulk relaxation (observed in the temporal dependence of adhesion-induced indentation upon tip extension) and relaxation at the tip–surface interface (observed in the temporal dependence of the retraction curve). The distinction between the mechanical response of a bulk polymer and its surface is one important goal of all point-probe measurements and has also been achieved by using dynamic INI [67].

#### Current status

Evidently a multitude of experiments and protocols are described in the literature for extracting the viscoelastic properties of soft polymers by using dynamic INI and AFM nanoindentation. The need to develop standard methods in dynamic nanoindentation has been shown to be crucial for the reliability and repeatability of the experiments. Such a standardized method should include a clear protocol for the modulation, the calibration process of the instrument, the reference materials and the frequency ranges. Some efforts have been made in this direction, but there is no consensus on the matter yet [51]. Nonetheless, it is anticipated that under the action of ISO TC164 SC3 accepted protocols will be decided upon [112].

When these two point probe techniques are used to measure the same surface, the divergence between INI and AFM is apparent. As indicated above, there are notable differences between what, and how they measure. The comparison between instrumented nanoindentation and AFM probe nanoindentation provided here has emphasized some of these differences.

#### Demonstration of the concepts

By way of demonstrating some of these issues, an experimental comparison between AFM and instrumented dynamic nanoindentation for assessing the viscoelasticity in polymers is presented below. Simple approaches were chosen to demonstrate that meaningful data can be obtained with relative ease, and also to point out the resultant inaccuracies. In order to highlight the issues that are discussed in this review, particular attention is given to the calibration of the instrument. The dynamic moduli of two polymers are estimated on the basis of Equation 12.

**Figure 5:**
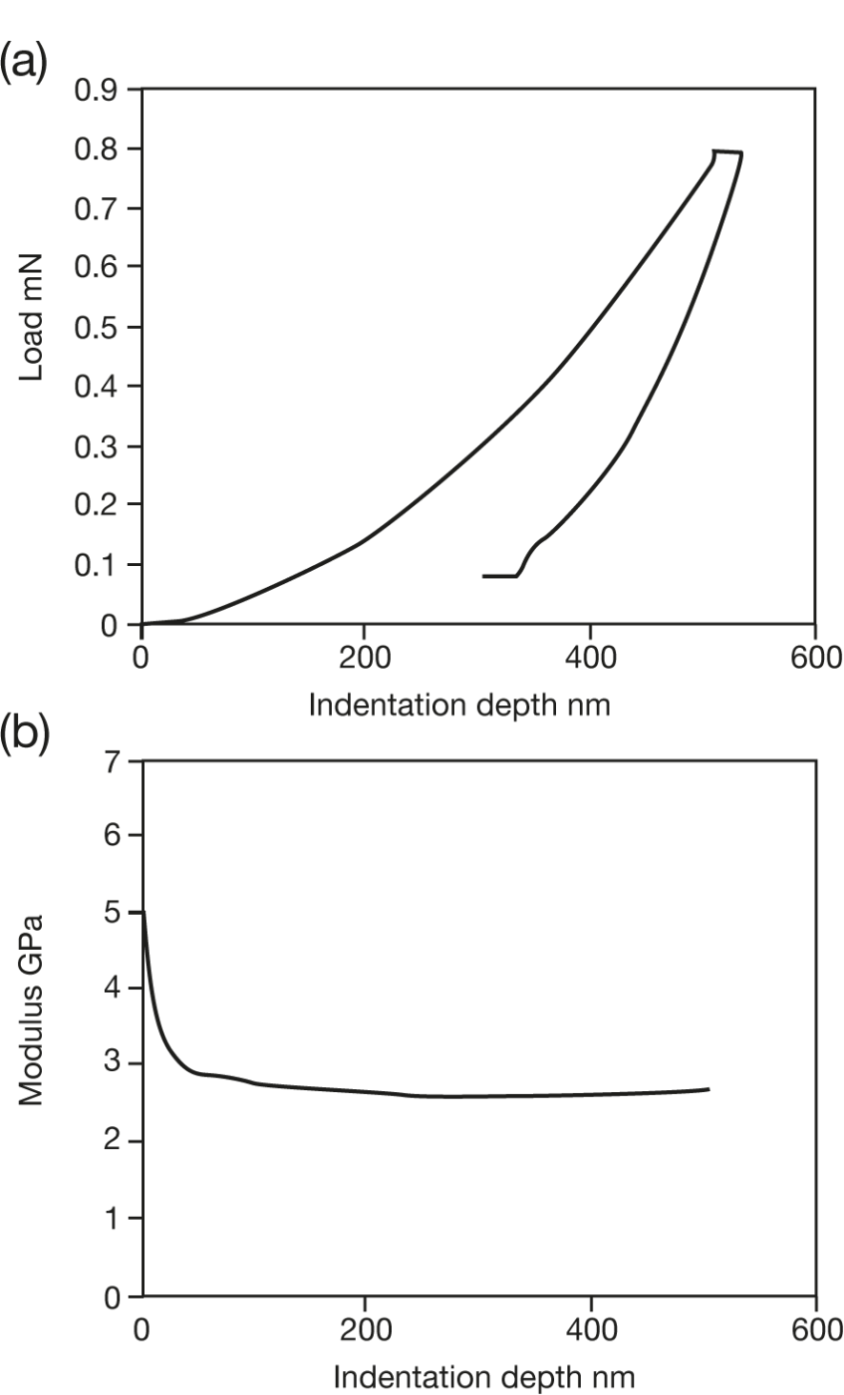
Modulus measurements on polypropylene (A) Load-vs-deformation curve (B) Reduced storage modulus determined using continuous stiffness measurement vs depth. CSM modulation amplitude 5 nm at 45 Hz.

Nanoindentation experiments were performed on isotactic polypropylene (iPP, 127,000 g/mol) by using instrumented nanoindentation (Agilent DCM) and AFM tip based nanoindentation (NTMDT NTEGRA). A typical INI load-vs-displacement curve is displayed in Figure 5A. The modulation results (continuous stiffness measurement CSM^TM^, modulation amplitude 5 nm) displayed in Figure 5B reflect the modulus value, which is determined by the instrument software by using standard O&P analysis [23]. The curve in Figure 5B starts at high *E* values and rapidly decreases down to an indentation depth of about 60 nm, after which it settles to an asymptotic value. This is due to surface effects in addition to the unreliability of the area function at very small depths and a relatively large amplitude of oscillation compared to the deformation at low depths. Averaged over 20 tests, the asymptotic reduced storage modulus is 2.7 ± 0.2 GPa. After adjusting for a Poisson ratio of 0.45, the resultant value of 2.15 GPa compares well with literature data that report a Young’s modulus of 1.8 GPa at this frequency [113]. The data in Figure 5 represent the storage modulus. In order to measure the smaller loss component, dynamic analysis was performed by using the relations summarized in this review, and reported below.

#### Dynamic instrumented nanoindentation

In order to extract the storage and the loss modulus from dynamic testing, the instrument response must be characterized and corrected for. The introduction of a frequency specific phase lock amplifier in the continuous stiffness measurement (CSM) induces a frequency-dependent phase shift. Consequently, a correction of the phase shift is required. The calibration is made according to the protocol described by Herbert et al. [64]: With the indenter tip hanging in free space the stiffness and the damping of the instrument are measured as a function of the frequency by using Equations 9–11 above.

**Figure 6:**
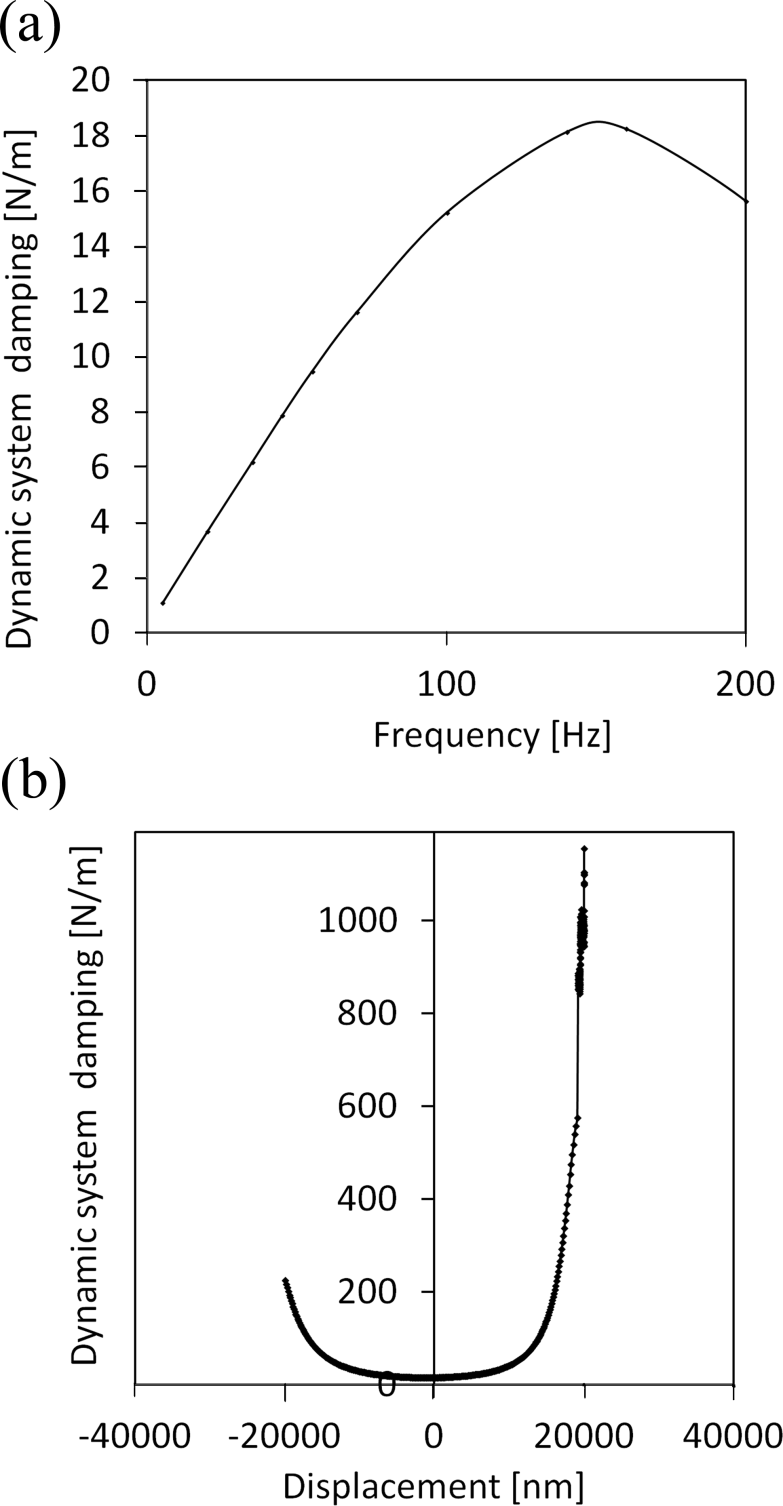
Calibration of the system damping for INI. The damping was computed for each frequency at different vertical extensions of the indenter over a range of ± 30 μm. The damping is computed by using Equations 9–11 and is reported here as *C*_i_ω. (a) System damping as a function of the frequency for a displacement near 0. (b) System damping as a function of the displacement of the indenter head at 200 Hz frequency.

The calibration revealed a significant variation in damping with the frequency (Figure 6).

**Figure 7:**
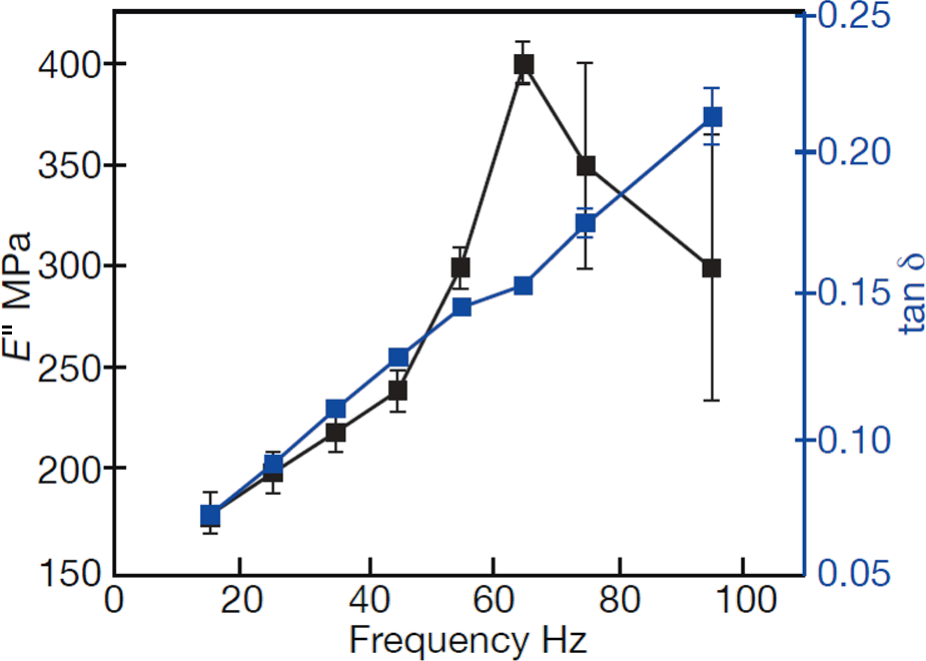
Dynamic INI of iPP as a function of the frequency. The error bars indicate the standard deviation calculated for at least five measurements in three different places.

Figure 7 shows the variation of the loss moduli and loss tangent with the modulation frequency (ω/2π) at a depth of 1000 nm for the iPP films. As mentioned previously, tan δ is particularly suited for detecting dissipative processes such as friction [60,114]. The progressive increase of the loss tangent with the frequency can be attributed to the increase of internal chain friction at higher frequencies [66]. In comparison to the storage modulus, the values of loss modulus observed in Figure 7 contribute little to the magnitude of the complex modulus, so the careful calibration procedure is essential to get accurate data.

#### AFM-based modulation

In this experiment, the tip was first indented to a depth of 50 nm into the sample in the AFM (Bruker, multimode). Prior to the experiment, the cantilever spring constant and the deflection sensitivity were determined with the Nanoscope software (former from the thermal noise and the latter by measuring the deflection of the cantilever with the displacement on a hard surface). The tip radius for various depths was estimated from a sample of known modulus by using the DMT relation and quantitative mechanical mapping (QNM^®^) mode (Bruker). A modulation was then applied at several frequencies in the range of 5–300 Hz. A digital lock-in amplifier was used to detect the amplitude and the phase of the cantilever oscillation relative to the drive signal. The modulation was conducted by using two oscillatory modes. In the first method, called *z*-modulation here, a sinusoidal signal is added to the *z*-voltage of the piezoelectric tube [99,115]. This leads to a modulated deflection of the cantilever of 5–10 nm and thus a variation of the force between tip and sample. This modulated amplitude is fed into a lock-in amplifier and the output of the lock-in amplifier is recorded as amplitude vs time. Because of the mechanical and instrumental response, the amplitude and phase of the instrumental contribution depend on the frequency. For a quantitative analysis of the data it is therefore necessary to correct for the response with the apparatus transfer function by using an incompressible sample. In the second mode, tip modulation, the cantilever base is modulated via the tip holder. Here the contribution of the instrument damping is isolated by vibrating the cantilever in air and the loss modulus of the sample is calculated by using Equation 11 and Equation 12.

**Figure 8:**
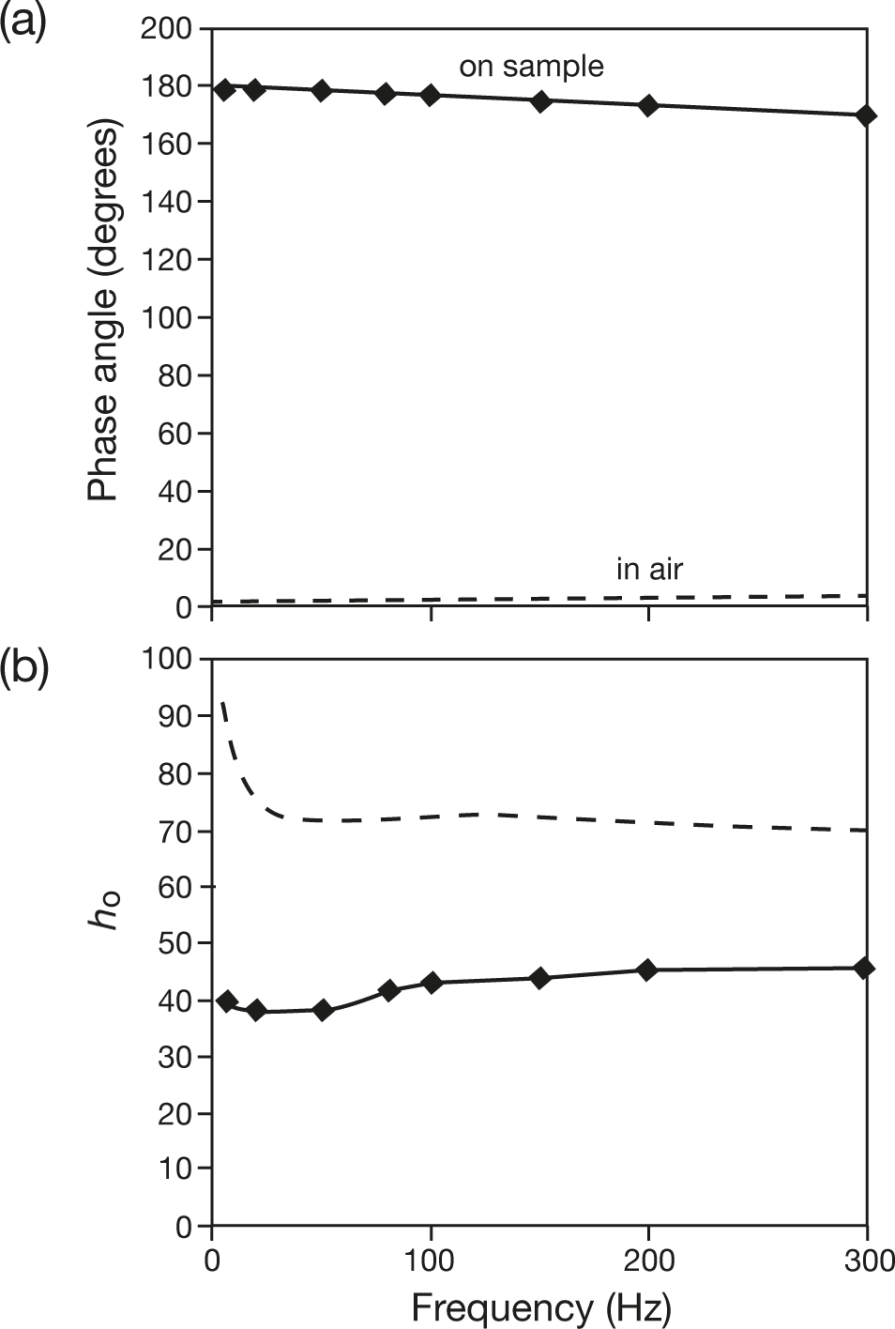
AFM tip modulation: (a) Phase shift and (b) amplitude in air (dashed line) and on sample (solid line).

The subtraction of instrumental damping is less straightforward in AFM than in INI. It has been suggested that for frequencies well below resonance, modulating the tip in air will give a negligible phase shift [102]. In our system, a small but observable shift can be detected as shown in Figure 8a. The phase lag rose slightly with the modulation frequency, from 0.5° at 5 Hz to 2.5° at 300 Hz. When the tip was brought in contact with the sample, the phase lag jumped to 180° as a direct consequence of the contact. After nulling this phase jump, the phase lag recorded on a hard surface was indeed larger than that recorded with the tip being suspended in air, and the phase lag increased from 2° to 10° in the frequency range from 5 to 300 Hz.

**Figure 9:**
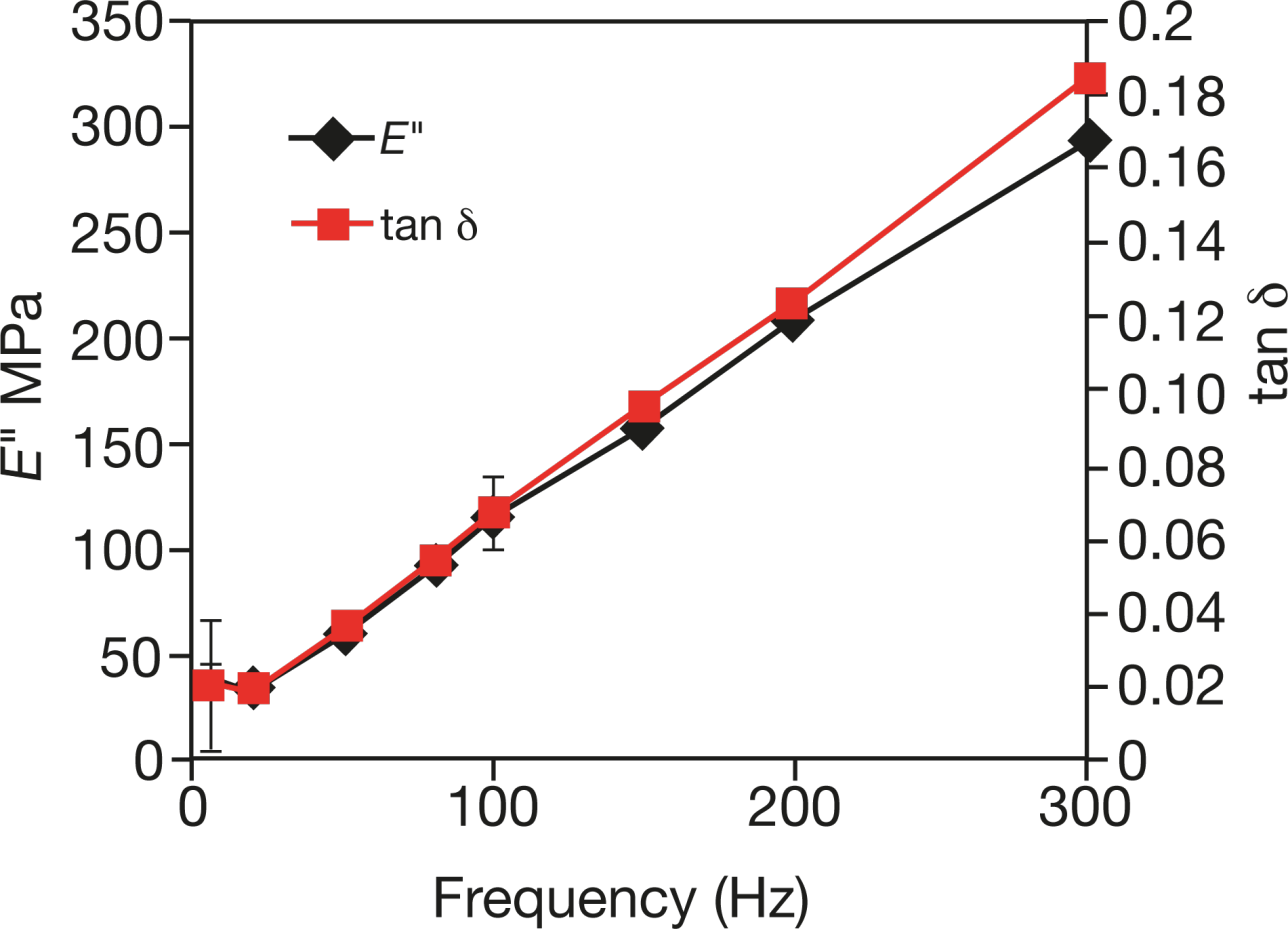
Dynamic AFM measurements of iPP as a function of the frequency. The measurements were made at a depth *h* = 50 nm by using a probe with a spring constant *k* = 33 N/m and a nominal tip radius of 50 nm. The oscillation amplitude was maintained at 10 nm.

Variations in the loss modulus and in tan δ with the modulation frequency were obtained from the AFM results for iPP according to Equation 12. The results are displayed in Figure 9. Increases of both the loss modulus and the loss tangent are observed. The comparison of both absolute values obtained in dynamic INI vs AFM and the change of E″ and tan δ with the frequency are favorable. The differences will be discussed below.

#### Creep measurements

Creep tests were performed on 1-mm thick polymethyl methacrylate (PMMA, *M*_w_ = 350,000 ) films. The tests were performed by using an AFM (NTEGRA, NT-MDT). The AFM probe was indented to an initial depth of 50 nm into the surface, then held at a constant load while monitoring the change in the *z*-sensor over a creep time of 10 s. The sensitivity of the detector was previously calibrated to compute the deformation variation with time *h*(*t*). During the creep test, the contact area between the tip and the sample increases with the displacement of the probe into the sample. The contact area is estimated as an approximately conical shape for depths of 25 nm and beyond, by using the manufacturer value for the half-angle of the tip.

The resulting curves were fit to Equation 13 to obtain *E*_∞_, the storage modulus at steady state conditions, as well as viscosity η_i_ and modulus *E*_i_ at characteristic times τ_i._ The latter is calculated from the relationship τ_i_ = η_i_/*E*_i_. Single, double, and triple exponential fits were attempted: the triple exponential fit was not significantly better than the double exponential fit. The following values are obtained: Asymptotic storage modulus, *E*_∞_ = 6.25 GPa; *E*_1_ = 1.53 GPa, η_1_ = 42 MPa·s, τ_1_ = 0.027 s; *E*_2_ = 2.08 GPa, η_2_ = 71 MPa·s, τ_2_ = 1.01 s.

**Figure 10:**
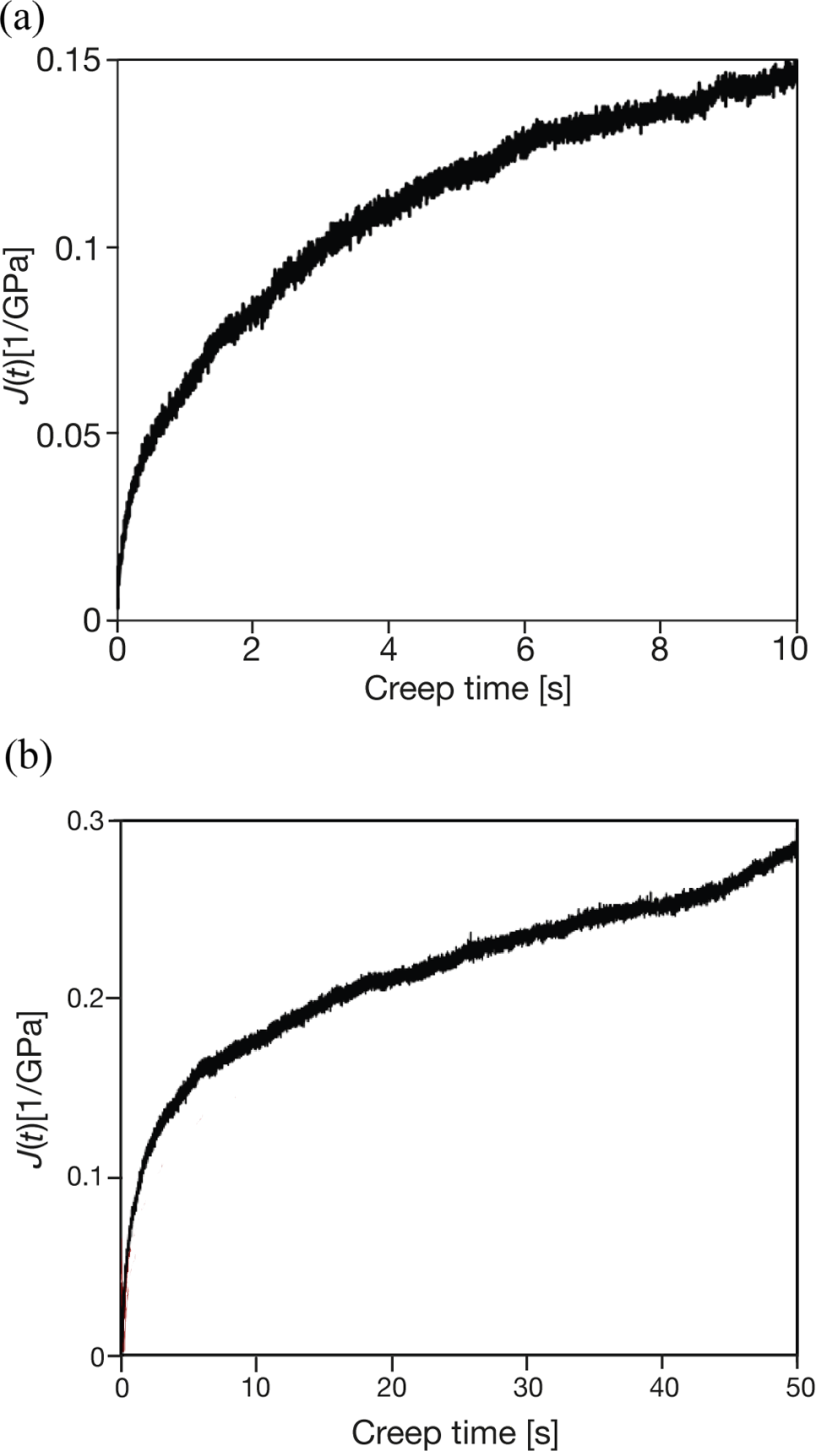
AFM probe creep test showing the creep compliance calculated using Equation 11 for PMMA. (a) A 10 second creep test. (b) Longer time showing effect of plastic yield (see text), in deviation of curve at long times.

As a comparison, the dynamic nanoindentation of PMMA gives *E*′ = 3.89 GPa and *E*″ = 0.537 GPa at 45 Hz and *E*’ = 3.4 GPa and *E*” = 0.33 GPa at 1 Hz. A recent study about the elastic and viscoelastic properties of PLLA/HA films that also used a biexponential fit found relaxation times of this order [116]. When the creep time is increased, plastic yielding occurs as seen in Figure 10. The graph deviates from the asymptotical behavior and *E*_∞_ increases (i.e., for *t* = 100 s, *E*_∞_ = 7.2 GPa).

## Discussion

The brief summary of the experimental analyses highlights the different factors that can influence the final results. The correlation between INI and AFM results should include a discussion of the dynamic testing protocol, surface effects, and calibration issues. In the following section, the data presented above will serve as a support for the comparison of the techniques, in terms of protocol and source of errors.

### Evaluation of the methods

#### Instrument calibration

In INI a calibrated force is applied along the surface normal. The load is controlled by electromagnetic or electrostatic actuators, the displacement is measured independently and the indenter head is supported by springs, in our case with a vertical stiffness of approximately 100 N·m^−1^ and a lateral stiffness of 10000 N·m^−1^ [64]. This strong asymmetry in compliance confines the motion to the surface normal in accordance with the Voigt model applied here. A key issue in dynamic nanoindentation is to successfully evaluate the instrumental contribution to the measured stiffness and damping. As previously noted, the frequency response of the instrument is controlled by the magnitude of the moving mass, the stiffness of the support springs and the damping of the head. Here INI has some advantages over AFM, for instance the widely used capacitive or electrostatic sensing of the indenter motion is stable over time and thus accurate for low frequencies, which allows for an access to frequencies usually used in rheological studies (0.1–1000 Hz).

Contrary to INI, among the various studies made on dynamic AFM, there is no consensus for quantifying the instrumental damping so that a number of different calibration methods exist. Most literature reports make use of a rigid substrate, such as sapphire or a silicon wafer as a reference sample. Since the substrate displays a purely elastic response, any phase shift observed can then be attributed to the instrument damping. As noted in [102], the use of a rigid sample is not accurate for materials that display small phase lags on the same order of that of the reference sample.

#### The modulation source

In the classical dynamic test at the macroscale the phase lag is measured from the strain response to a sinusoidal tensile/flexural stress. In contrast the phase lag in nanoindentation is controlled by the displacement response of the indenter to sinusoidal loading. The combined response of the instrument and the sample is described in the case of dynamic INI by two springs in parallel. The parallel model describes a configuration, in which equal forces are applied on both sample and indenter tip. This is in line with AFM, where a displacement, rather than a force is applied, typically at the base of the cantilever so the two springs (sample and instrument) are in series. The fact that the force is not restricted to the surface normal in AFM has been discussed above and also widely in the literature [63–64,117]. Lateral forces can arise because of the angle between the cantilever and the sample owing to the instrumental configuration. But this can be compensated for by controlling the angular path of the loading curve [118].

Even with such a correction the cantilever may twist during the indentation, especially at the start of the indentation, because of a torque arising from the imperfect vertical loading of the tip owing to the sample roughness, and the alignment of sample or tip. This can lead to either a flexural or a torsional force. The former will be detected in an optical-lever-based AFM system as normal force. The latter may, or may not, influence the detected normal force signal depending on the extent of cross-talk, but it will certainly influence the overall force balance. The torsional force could be significant in magnitude, considering the much larger magnitude of the torsional spring constant, which for a rectangular cantilever is related to the normal spring constant with a proportionality constant of [cantilever length/tip height]^2^. For typical Si single crystal micromachined cantilevers this factor ranges between 30–1000 [44]. Edwards et al. developed a correction factor for both rectangular and v-shaped cantilevers, which encompasses the tilt and the torque of the cantilever and extracts the pure normal force [119]. The correction also accounts for different tip geometries.

#### Surface effect-surface detection

The ability of these nanoscale techniques to probe the outer few nm of the surface presents an opportunity and a challenge: Firstly, a proper location on the surface requires great precision. The pitfalls in improper surface detection have been highlighted by Deuschle et al. [120]. In that work, the detection of the surface by triggering on a rise in the force above a noise threshold was compared to changes in contact stiffness that were derived from the amplitude of the modulation, *P*_0_/*h*_0_. For a PDMS sample with a modulus of 1 MPa, the surface was "missed" by 580 nm when using the former method with a resulting error in the computation of the modulus of 400%, whereas for a dynamic approach the overshoot was only 30 nm and the error in modulus only 10%. Various extrapolation methods can be used to determine the contact point [41,121–122] and it may be done by fitting the approach curves to an appropriate model [34,123]. Surface effects dictate the mechanical response in many nanoscale problems. Furthermore, surface properties can be quite different than those of the bulk. On the other hand, limiting the analyses to only a few nm presents a few more problems – firstly, discrete atomic/molecular events may be important, and the models that are used in general and are covered in this review are all continuum models. Secondly, getting knowledge of the contact geometry is much more difficult at this scale: Presumptions of a smooth surface and a geometrically ideal axiosymmetric indenter do not hold. In addition, the effects already discussed, which involve the influence of adhesion, etc., become dominant at this scale. For example, one dynamic study on an epoxy surface found that it was necessary to penetrate the surface by 130 nm in order to overcome the surface roughness and get representative results [124]. Monitoring the convergence of the CSM values with depth as shown in Figure 5 helps to estimate the depth at which surface geometric effects are overcome. Recently, accurate measurements of the modulus for several polymers at depths of only several nanometers was shown to be possible by properly accounting for adhesion, carefully characterizing the tip shapes, and limiting the maximum stress [125]. Surface effects also influence the models chosen, both because the linear elastic regime is exceeded at relatively small loads and depths, and because the tip geometry is hard to track over the different size scales. Sometimes, this depth cannot be controlled, for instance when the adhesive force is large (see Figure 1) and even at zero external loading force the depth can be significant.

#### Sources of error and internal calibration

Internal calibration and errors are perhaps the most critical aspect of nanoindentation. Here too, there is a divergence between the emphasis in AFM and in INI.

#### Spring stiffness and instrument compliance

For INI the force is applied directly to the tip through a calibrated transducer: This force is the same as that on the sample. Thus, over a wide range of sample moduli, the stiffness of the spring is independent of the stiffness of the studied material. In contrast, AFM is controlled by displacement. The total displacement is split into cantilever bending and deformation, so that the ultimate force that can be applied on the sample is dependent on the cantilever spring stiffness [126]. The choice of a particular cantilever fixes the spring constant and hence ultimate deformation of the sample. If the deformation is too small, there will be sensitivity problems, and if it is too large, the assumptions on contact area, deformation mode, and even linear elasticity may be violated. In general, one cantilever probe can test a limited (approximately 2 orders of magnitude) span of elastic moduli. A useful rule of thumb for choosing an appropriate spring may be estimated from the contact stiffness for a Hertzian contact *S* = 2aE^*^ [20].

There are a number of ways to calibrate the cantilever spring constant, covered in a review by Sader [127], and even careful calibration will generally result in a relative uncertainty on the order of 10%. The forces in INI are generally factory calibrated, although most manufacturers provide for some on-site calibration. Standard procedures for good working practice strongly recommend 1% tolerance for both force and displacement [128]. Instrument compliance must also be included in the determination of the sample deformation. For polymers that have elastic moduli of a few GPa or less, the precise calibration of this value is not expected to contribute significantly to the results.

#### Contact area

A main shortcoming of nanoindentation is the inability to optically view the indentation area in real time, or directly upon load release as in microindentation. Rather, the contact area is calculated based on the knowledge of the accurate indenter geometry, which in turn yields the projected contact area at each depth. This is only possible for a well-defined tip. The INI community developed several methods to accurately estimate the shape of the tip. One method is based on an AFM scan of the tip, which gives the real geometry of the indenter [129]. But this requires removing the tip from the head for each such measurement. For small depths, as they are used in nanoindentation, the shape at the end of the tip is critical and this geometry can change significantly over the course of a day’s work [130–131]. Some attempts have also been made to image the imprint of the indenter left in the sample by AFM scanning [57,129]. Another possibility is to use the INI indenter tip as a probe scanner to scan a sample with very sharp features as it is widely done for AFM tip radius calibration [132–133].

Blind reconstruction is attractive since it only requires the data obtained from the topographical scan that was performed during the routine course of the experiment [132]. However, this technique does not give the complete area function of the tip, rather only for those parts that come into contact with the sample during such a scan [134]. When deeper depths are accessed in the indentation cycle, the relevant data needed about the tip profile may not be available. For this reason, reference-based techniques have become attractive, in a fashion quite similar to that adapted in the O&P method for INI, in which a standard sample of known modulus is used to estimate the tip shape in AFM [134–135]. Recently, AFM tip areas were determined by first applying a large tip that could be characterized optically using INI, then using that value as a reference for determining the AFM tip size by Hertzian analysis [136]. The objective of tip-shape calibration is to estimate the cross-section area of the indenter tip as a function of the distance from the apex. Although most procedures make such tip calibration on one sample, calibrating the area function on two different materials (for instance, sapphire and fused quartz) further increases the accuracy of the area function [122].

In contrast to INI, AFM probes are consumables and often several tips may be used in the course of a measurement, so extensive calibrations are not practical. One should keep in mind that those calibrations still depend on the reliability of the internal calibration (deflection sensitivity, spring constant, validity of analytical model, topography). They also depend on the reliability of the analytical model used to back-calculate the tip area from a known modulus. In this context, it is worth noting that the determination of the loss factor, tan δ, does not require a determination of the area thus removing a major source of uncertainty [137].

#### Deflection sensitivity

The force applied to the cantilever results in a flexure, characterized by the deflection angle in an optical beam setup. The deflections must be kept within the linear response regime of both cantilever spring and optical system. This linear response is calibrated by pressing the tip on a non-deforming surface. Although simple to perform, care is required to obtain a meaningful result: The displacement calibration can vary with the tip velocity [138] and the alignment of the cantilever. The calibration is also prone to artifacts, such as those due to friction at the tip–surface contact that can introduce systematic errors to the value [139].

## Conclusion

This review has outlined the considerations which should be made in the nanomechanical testing of viscoelastic materials by using point probes. It should be clear from the reading that there are still many loose ends to tie up in order to discover the best way to make such measurements. However, both the INI and AFM techniques have the means and technology in place to push the field forward. Great strides have been made in ensuring the reliability and usefulness of the data. The advances, both on the technical and the conceptual level, thus work in unison to forge new levels of understanding of viscoelastic processes at the nanoscale.
